# Modulation of liver regeneration via myeloid PTEN deficiency

**DOI:** 10.1038/cddis.2017.47

**Published:** 2017-05-25

**Authors:** Wen-Tao Ma, Yan-Jie Jia, Qing-Zhi Liu, Yan-Qing Yang, Jing-Bo Yang, Zhi-Bin Zhao, Zhen-Ye Yang, Qing-Hua Shi, Hong-Di Ma, M Eric Gershwin, Zhe-Xiong Lian

**Affiliations:** 1 Liver Immunology Laboratory, Institute of Immunology, Hefei, China; 2 The CAS Key Laboratory of Innate Immunity and Chronic Disease, School of Life Sciences, University of Science and Technology of China, Hefei, China; 3 Division of Rheumatology, Allergy and Clinical Immunology, University of California at Davis School of Medicine, Davis, CA, USA; 4 Innovation Center for Cell Signaling Network, Hefei National Laboratory for Physical Sciences at Microscale, Hefei, China

## Abstract

Molecular mechanisms that modulate liver regeneration are of critical importance for a number of hepatic disorders. Kupffer cells and natural killer (NK) cells are two cell subsets indispensable for liver regeneration. We have focused on these two populations and, in particular, the interplay between them. Importantly, we demonstrate that deletion of the myeloid phosphatase and tensin homolog on chromosome 10 (PTEN) leading to an M2-like polarization of Kupffer cells, which results in decreased activation of NK cells. In addition, PTEN-deficient Kupffer cells secrete additional factors that facilitate the proliferation of hepatocytes. In conclusion, PTEN is critical for inhibiting M2-like polarization of Kupffer cells after partial hepatectomy, resulting in NK cell activation and thus the inhibition of liver regeneration. Furthermore, PTEN reduces growth factor secretion by Kupffer cells. Our results suggest that targeting PTEN on Kupffer cells may be useful in altering liver regeneration in patients undergoing liver resection.

Liver regeneration is the compensatory hyperplasia of the liver in response to injury. During this process, the innate immune system, particularly Kupffer cells and natural killer (NK) cells, has a fundamental role. Several lines of evidence suggest that Kupffer cells support liver regeneration, particularly based on secretion of tumor necrosis factor alpha (TNF-*α*) and interleukin (IL)-6, which facilitate activation of signal transducer and activator of transcription 3 (STAT3) in hepatocytes.^1, 2^ Kupffer cells are also critical for initiating the proliferation of liver progenitor cells following liver injury.^3^ In contrast, NK cells negatively modulate liver regeneration.^4^ This effect appears secondary to the secretion of interferon-gamma (IFN-*γ*), which activates STAT1 and antagonizes STAT3 activation in hepatocytes. Interestingly, NK cells are the most important source of IFN-*γ* after partial hepatectomy (PHx). Moreover, a recent study suggests that deficiency of the co-inhibitory receptor TIGIT on NK cells leads to overactivation and thus potentially impedes liver regeneration.^5^

The phosphatase protein, phosphatase and tensin homolog on chromosome 10 (PTEN), was originally identified as a tumor-suppressor protein, and is commonly mutated or deleted in a wide variety of tumors.^6, 7^ PTEN is a lipid phosphatase that can negatively modulate the phosphatidylinositol 3 kinase (PI3K)-Akt signaling pathway, one of the most important drivers of cell survival and proliferation.^8, 9^ In addition, PTEN is also a positive regulator of TLR4 signaling in murine peritoneal macrophages, partly through suppression of the mitogen-activated protein kinase (MAPK) signaling pathway.^10^ PTEN can also regulate the expression of several genes required for M2 polarization in peritoneal macrophages and modulate inflammatory cytokine production in the liver.^11, 12, 13^ Nevertheless, the role of PTEN in Kupffer cells is elusive, and PTEN involvement in the process of liver regeneration is unclear.

We propose that a better understanding of the interplay of Kupffer cells and NK cells is essential to understand the molecular events that modulate liver regeneration. In support of this hypothesis, we demonstrate that myeloid PTEN deficiency results in an M2-like polarization of Kupffer cells, which are less able to activate NK cells and thus alter regeneration. Indeed, PTEN deficiency also enhances production of growth factors by Kupffer cells. In conclusion, our data highlight a novel molecular mechanism that controls Kupffer cell phenotype and Kupffer cell–NK cell interactions during liver regeneration. This study may provide a potential target for promoting improved liver regeneration following liver resection.

## Results

### Characteristics of liver Kupffer cells after PHx

**Figure 1:**
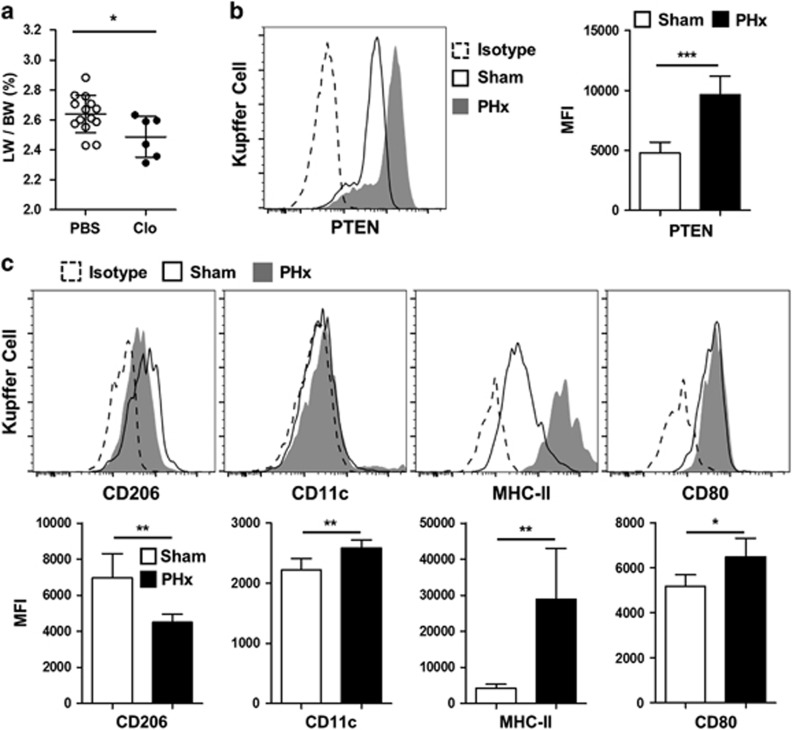
Liver Kupffer cells show upregulated PTEN expression level and an M1-like polarization after 2/3 PHx. (**a**) The liver weight (LW) to body weight (BW) ratio was analyzed 48 h post-2/3 PHx in PBS-liposome-treated (PBS, *n*=14) or clodronate-liposome-treated (Clo, *n*=6) mice. (**b**) PTEN expression levels in CD45^+^F4/80^hi^CD11b^lo^ Kupffer cells of sham-operated (*n*=5) or 24 h PHx-treated mice (*n*=5) were analyzed using flow cytometry. (**c**) Expression levels of CD206, CD11c, MHC-II and CD80 were analyzed in Kupffer cells from sham (*n*=5) or 24 h PHx-treated mice (*n*=5) using flow cytometry

Kupffer cells were depleted using clodronate liposomes (Supplementary Figures 1A–C), which significantly compromised the liver regeneration rate (*P*=0.0264, Figure 1a). Moreover, as peritoneal macrophages were reported to modulate liver repair during sterile inflammation,^14^ the peritoneal cavity was washed with PBS 24 h before 2/3 PHx. Meanwhile, these mice were treated with PBS-liposomes or clodronate liposomes, respectively. The result showed a similar trend, as Kupffer cell depletion in these peritoneal cavity washed mice also significantly compromised the liver regeneration rate (*P*=0.0364, Supplementary Figure 1D). Furthermore, the PTEN expression level increased markedly in Kupffer cells after 2/3 PHx (*P*=0.0003, Figure 1b), whereas Kupffer cell numbers did not change significantly after PHx (Supplementary Figure 1E). Instead, the increase in PTEN level was accompanied by a more activated M1-related phenotype of Kupffer cells, as reflected by the downregulation of CD206 (*P*=0.0058), and upregulation of CD11c (*P*=0.0086), MHC-II (*P*=0.0019) and CD80 (*P*=0.0170) (Figure 1c).

### PTEN^mKO^ mice show a more prominent liver-regenerating capacity after PHx

**Figure 2:**
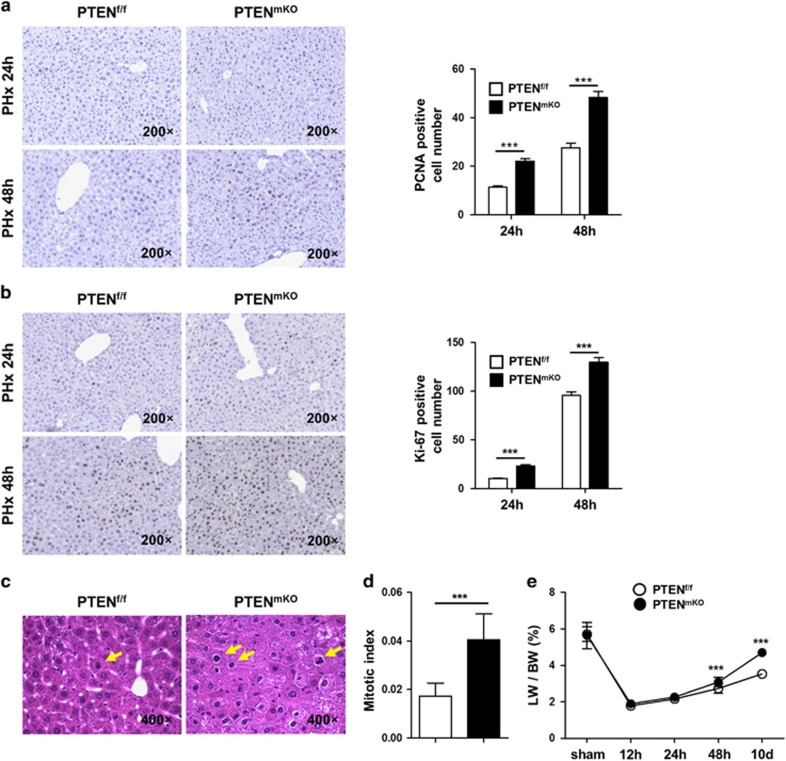
PTEN^mKO^ mice show a more prominent liver-regenerating capacity after 2/3 PHx. (**a**) Immunohistochemical staining of PCNA in liver sections from PTEN^f/f^ (*n*=32 for 24 h, *n*=16 for 48 h) and PTEN^mKO^ (*n*=30 for 24 h, *n*=20 for 48 h) mice 24 and 48 h after 2/3 PHx. PCNA-positive hepatocytes were counted in five randomly chosen fields, and the average count of these five fields was set as the PCNA-positive cell number of one mouse. (**b**) Immunohistochemical staining of Ki-67 in liver sections of PTEN^f/f^ (*n*=29 for 24 h, *n*=15 for 48 h) and PTEN^mKO^ (*n*=27 for 24 h, *n*=20 for 48 h) mice 24 and 48 h after 2/3 PHx. The method for counting Ki-67-positive cells was the same as in **a**. (**c** and **d**) Representative H&E staining (**c**) and quantification (**d**) of mitotic hepatocytes (indicated by yellow arrows) of PTEN^f/f^ (*n*=8) and PTEN^mKO^ mice (*n*=7). The mitotic index was calculated as the average ratio of mitotic hepatocytes (hepatocytes in prometaphase, metaphase and anaphase) to total hepatocytes of five randomly chosen fields. (**e**) The liver weight (LW) to body weight (BW) ratio was calculated at various time points in sham- or PHx-operated PTEN^f/f^ (*n*=3 for sham, *n*=4 for 12 h, *n*=5 for 24 h, *n*=8 for 48 h, *n*=5 for 10 days) and PTEN^mKO^ (*n*=4 for sham, *n*=6 for 12 h, *n*=4 for 24 h, *n*=8 for 48 h, *n*=5 for 10 days) mice

We further used LysM^cre/+^PTEN^f/f^ mice (PTEN^mKO^) and littermate PTEN^f/f^ mice following PHx to investigate the role of PTEN in Kupffer cells during liver regeneration. There was no significant difference in liver injury between PTEN^mKO^ and PTEN^f/f^ mice (Supplementary Figures 2A and B). Nevertheless, compared with PTEN^f/f^ control mice, PTEN^mKO^ mice showed an enhanced liver regeneration rate, as indicated by immunohistochemical staining of PCNA (*P*<0.0001 for 24 and 48 h) and Ki-67 (*P*<0.0001 for 24 and 48 h) in liver tissues (Figures 2a and b). In addition, hepatocytes of PTEN^mKO^ mice demonstrated a more rigorous mitosis rate (*P*=0.0001, Figures 2c and d, and Supplementary Figure 3A). The ratio of liver weight to body weight in PTEN^mKO^ mice was also much higher compared with PTEN^f/f^ mice 48 h post PHx (*P*<0.0001 for 48 h and 10 days, Figure 2e).

### PTEN-deficient Kupffer cells demonstrate an M2-like polarization state

**Figure 3:**
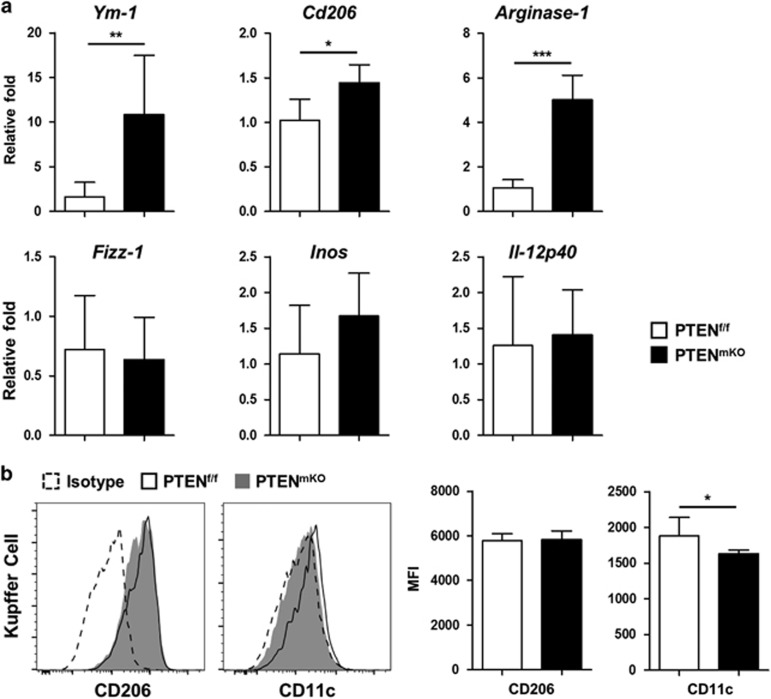
PTEN-deficient Kupffer cells show an M2-like polarization state. (**a**) Real-time PCR analysis of M2-related (*Ym-1*, *Cd206*, *Arginase-1* and *Fizz-1*) and M1-related (*Inos* and *Il-12p40*) markers in collagenase-perfused Kupffer cells from 48 h PHx-operated PTEN^f/f^ (*n*=6) and PTEN^mKO^ (*n*=6) mice. (**b**) CD206 and CD11c expression levels in CD45^+^F4/80^hi^CD11b^lo^ Kupffer cells from PTEN^f/f^ (*n*=6) and PTEN^mKO^ (*n*=4) mice 48 h post PHx were analyzed using flow cytometry

PTEN deficiency did not alter CD206 (M2) and CD11c (M1) expression levels of liver monocyte-derived macrophages (MoDMs), which expressed differential levels of Ly-6C and no Ly-6G (Supplementary Figures 1B and 4A). However, liver resident Kupffer cells from PTEN^mKO^ mice exhibited an M2-like polarization state in steady state (*P*=0.0016 for *arginase-1*, *P*=0.0398 for *fizz-1* and *P*=0.0075 for CD11c, Supplementary Figures 5A and B) and after PHx (*P*=0.0072 for *ym-1*, *P*=0.0041 for *arginase-1* and *P*=0.0120 for *cd206* by real-time PCR, and *P*=0.0147 for CD11c by flow cytometry; Figures 3a and b) compared with PTEN^f/f^ mice. In addition, PTEN^mKO^ mice showed significantly higher levels of phosphorylated Akt and FoxO1 compared with PTEN^f/f^ mice (*P*=0.0480 for p-Akt and *P*=0.0003 for p-FoxO1, Supplementary Figure 6). Considering that activated Akt signaling and thus inhibited FoxO1 signaling was involved in suppressing proinflammatory cytokine secretion and promoting M2-like polarization of macrophages,^13, 15^ this result indicated that PTEN may regulate the polarization of Kupffer cells through downstream Akt/FoxO1 signaling pathway.

### NK cells from PTEN^mKO^ mice are less activated owing to fewer activation signals from Kupffer cells

**Figure 4:**
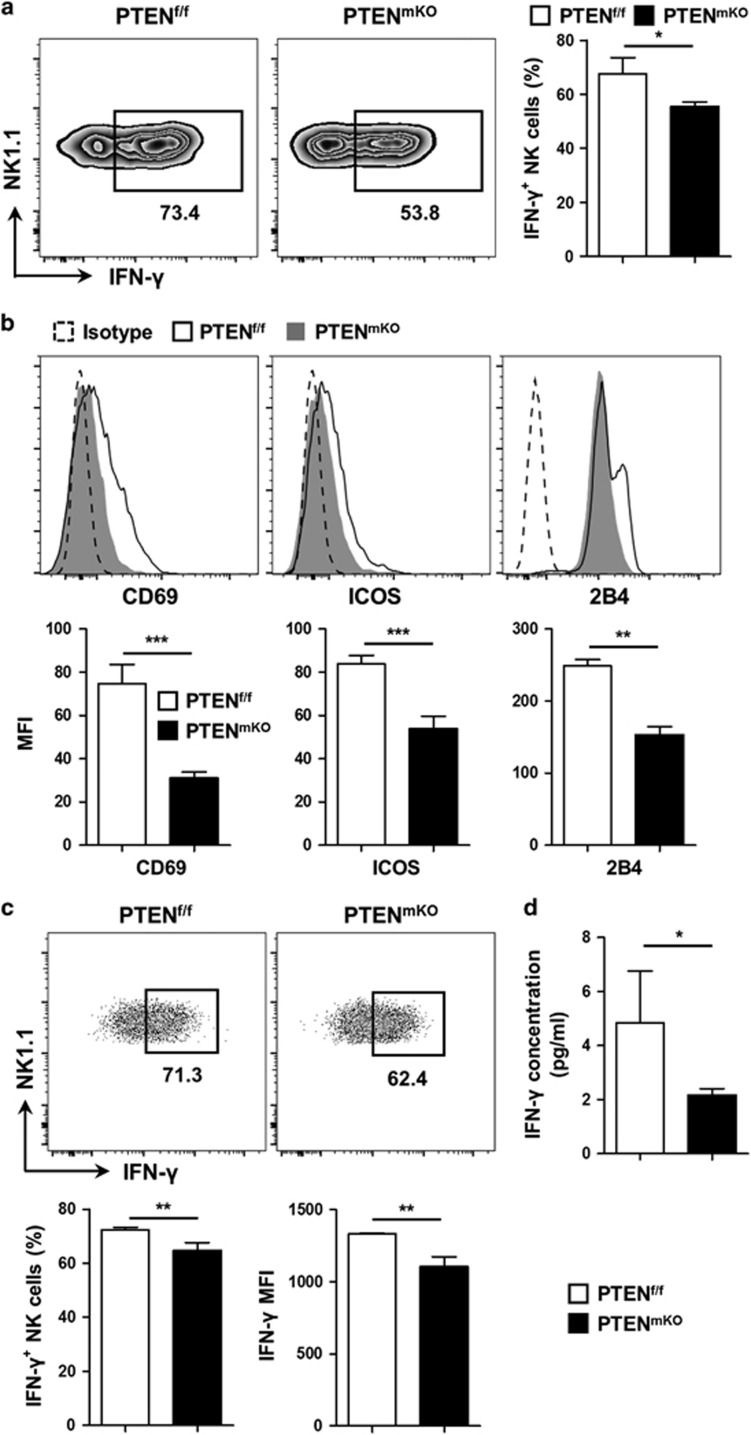
NK cells from PTEN^mKO^ mice are less activated. (**a**) Total liver lymphocytes from PTEN^f/f^ (*n*=6) and PTEN^mKO^ (*n*=4) mice were isolated 48 h post PHx, and treated with a PMA/ionomycin cocktail for 4 h. The fraction of IFN-*γ*-positive NK cells (CD3^-^NK1.1^+^) was analyzed using flow cytometry. (**b**) The mean fluorescence intensity (MFI) of CD69, ICOS and 2B4 on NK cells from PTEN^f/f^ (*n*=5) and PTEN^mKO^ (*n*=5) mice 48 h post PHx was analyzed by flow cytometry. (**c**) FACS-sorted liver Kupffer cells from PTEN^f/f^ (*n*=4) or PTEN^mKO^ (*n*=4) mice 48 h post PHx were co-cultured with sorted WT liver NK cells at a ratio of Kupffer: NK=1:2, in the presence of 50 IU/ml IL-2. The percentage of IFN-*γ*-positive NK cells and the mean fluorescence intensity of IFN-*γ* were analyzed using flow cytometry after 48 h of co-culture. (**d**) The IFN-*γ* concentration in the medium of the co-culture described in **c** was measured by cytometric bead array

NK cells from PTEN^mKO^ mice were less activated after PHx, as reflected by their decreased ability to secrete IFN-*γ* (*P*=0.0133, Figure 4a) and reduced levels of activation-associated surface markers (*P*<0.0001 for CD69, *P*<0.0001 for ICOS and *P*=0.0063 for 2B4, Figure 4b). In contrast, the NK cell activation state was similar between sham-operated PTEN^mKO^ mice and control mice (Supplementary Figure 7). In addition, NK cell proliferation and apoptosis profiles were not affected by myeloid PTEN deficiency after PHx (Supplementary Figures 8A–D). Moreover, the *in vitro* co-culture experiment demonstrated that NK cells co-cultured with PTEN^mKO^ mice-derived Kupffer cells had suppressed IFN-*γ* secreting ability (*P*=0.0079 for IFN-*γ* percentage, *P*=0.0025 for IFN-*γ* MFI; Figure 4c). The IFN-*γ* concentration was also lower in the culture supernatants of NK and PTEN^mKO^ Kupffer cell co-culture (*P*=0.0316, Figure 4d).

**Figure 5:**
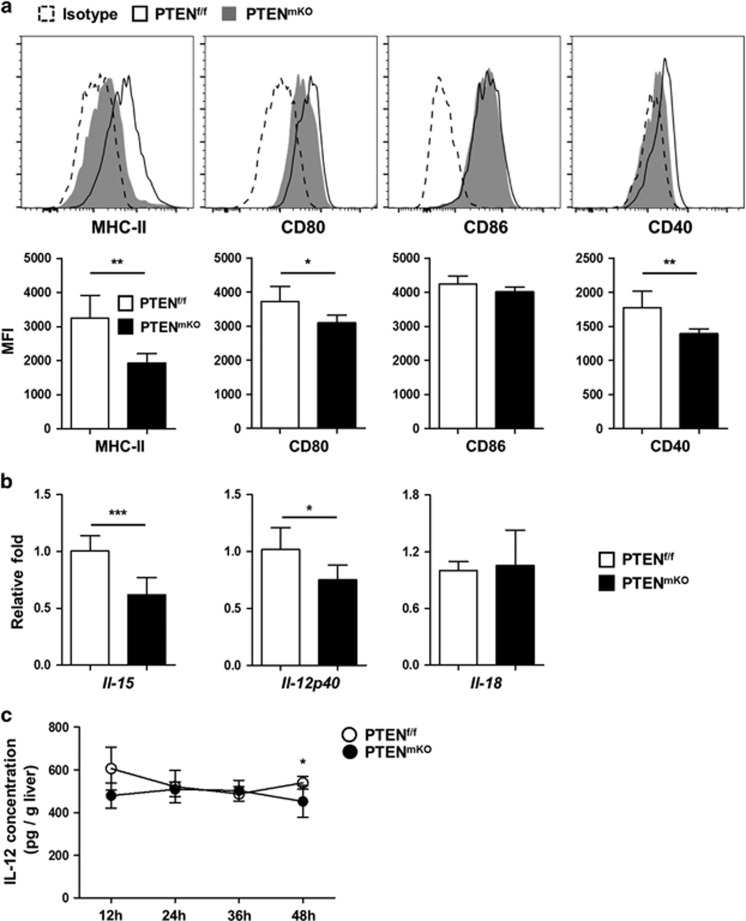
Kupffer cells from PTEN^mKO^ mice express less NK cell-activating factors. (**a**) Livers of PTEN^f/f^ (*n*=6) and PTEN^mKO^ (*n*=4) mice 48 h post PHx were perfused by collagenase *in situ*, and Kupffer cells were analyzed for the expression levels of MHC-II, CD80, CD86 and CD40 by flow cytometry. (**b**) Livers of PTEN^f/f^ (*n*=5) and PTEN^mKO^ (*n*=5) mice 3 h post PHx were perfused to isolate Kupffer cells, and the expression levels of *Il-15*, *Il-12p40* and *Il-18* were analyzed by real-time PCR. (**c**) Livers of PTEN^f/f^ (*n*=6 for 12 h, *n*=5 for 24 h, *n*=5 for 36 h and *n*=6 for 48 h) and PTEN^mKO^ (*n*=6 for 12 h, *n*=5 for 24 h, *n*=5 for 36 h and *n*=6 for 48 h) mice were homogenized in PBS at various time points post PHx, and analyzed by CBA for IL-12 concentration

PTEN^mKO^ Kupffer cells, but not MoDMs, also displayed lower levels of MHC-II (*P*=0.0060), CD80 (*P*=0.0316) and CD40 (*P*=0.0094) (Figure 5a and Supplementary Figure 9A), which correlated directly or indirectly with NK cell activation. Moreover, levels of the NK cell-activating cytokines *il-15* (*P*=0.0009) and *il-12p40* (*P*=0.0333) levels were shown to be significantly decreased in PTEN^mKO^ Kupffer cells compared with those of PTEN^f/f^ mice by real-time PCR (Figure 5b). CBA analysis confirmed that IL-12 level was significantly decreased in livers of PTEN^mKO^ mice compared with those of PTEN^f/f^ mice at 48 h post PHx (*P*=0.0229, Figure 5c). Further experiments were conducted to investigate the significance of direct cell–cell contact of Kupffer cells and NK cells and indirect cytokine production from Kupffer cells in the regulation of NK cell activation. The result turned out that both manner were necessary in this process, as both Transwell treatment and IL-12p40-deficient Kupffer cells showed significantly compromised NK cell-activating capacity (*P*<0.0001 for NK+PTEN^f/f^ Kupffer cells, *P*=0.0204 for NK+PTEN^mKO^ Kupffer cells and *P*=0.0002 for WT vs p40^−/−^ Kupffer cells co-cultured with NK cells, Supplementary Figure 10).

### PTEN-deficient Kupffer cells are more mitogenic for hepatocytes

**Figure 6:**
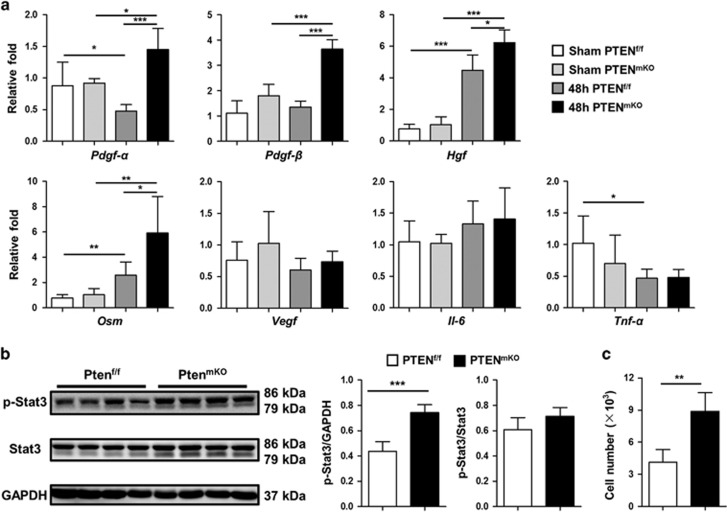
PTEN-deficient Kupffer cells are more mitogenic for hepatocytes. (**a**) To isolate Kupffer cells, sham and 48 h PHx-operated livers of PTEN^f/f^ (*n*=7 for sham, *n*=6 for 48 h) and PTEN^mKO^ (*n*=5 for sham, *n*=6 for 48 h) mice were perfused by collagenase *in situ*, and the expression levels of hepatocyte mitogenic related factors (*Pdgf-α, Pdgf-β, Hgf, Osm, Vegf, Il-6* and *Tnf-a*) were analyzed using real-time PCR. (**b**) Total liver proteins from PTEN^f/f^ and PTEN^mKO^ mice were extracted 48 h after PHx. Levels of p-Stat3, Stat3 and GAPDH were evaluated by western blotting (left), and levels of p-Stat3 (relative to GAPDH and total Stat3) were analyzed with ImageJ (right). (**c**) FACS-sorted liver Kupffer cells from PTEN^f/f^ (*n*=5) or PTEN^mKO^ (*n*=5) mice 48 h post PHx were cultured alone for 24 h, and AML-12 cells were stimulated with the Kupffer cell conditioned medium for 48 h. Then, AML-12 cells were dissociated with Trypsin-EDTA and counted

Interestingly, although the levels of growth factors were similar in sham-operated PTEN^f/f^ mice and PTEN^mKO^ mice, they increased markedly in response to PHx in both groups of mice. Most importantly, the levels of *pdgf* (*P*<0.0001 for pdgf-*α* and *P*<0.0001 for pdgf-*β*), *hgf* (*P*=0.0177) and *osm* (*P*=0.0261) were all significantly higher in PHx-treated PTEN^mKO^ mice compared with PTEN^f/f^ control mice (Figure 6a). In accordance with this, significantly higher levels of phosphorylated Stat3 (p-Stat3), a downstream signaling molecule, were observed in PTEN^mKO^ mice 48 h post PHx (*P*=0.0008) (Figure 6b). This direct mitogenic role of Kupffer cell was corroborated *in vitro*; conditioned medium from PTEN^mKO^ mouse-derived Kupffer cells more potently stimulated mouse hepatocyte cell line growth (*P*=0.0014, Figure 6c).

## Discussion

PTEN deletion affects the polarization of peritoneal macrophages.^11, 12^ Nevertheless, the characteristics and functions of macrophages vary depending on the specific microenvironment.^16^ Our current data demonstrate that PTEN deletion in myeloid cells facilitates the M2-like polarization of Kupffer cells and increases the rate of liver regeneration. Considering the supportive role of Kupffer cells in liver regeneration,^1, 2^ the upregulation of PTEN in Kupffer cells may function as a negative regulator of liver regeneration after PHx. Thus, it is possible that Kupffer cells are not always helpful for liver regeneration, but also functions to negatively modulate this process to prevent over-regeneration.

As previous investigations demonstrated, M lysozyme gene was not specifically expressed by Kupffer cells, and this gene was highly expressed by neutrophils, monocytes, macrophages and partially expressed by dendritic cells.^17^ However, the conclusion of this study could be supported by several results. First foremost, as shown by our results, the NK cell-activating ability of sorted PTEN^mKO^ Kupffer cells was less strong compared with PTEN^f/f^ Kupffer cells (Figure 4), and sorted PTEN^mKO^ Kupffer cells stimulated the proliferation of hepatocytes more potently *in vitro* (Figure 6). In these two parts of *in vitro* experiments, the Kupffer cells we used were sorted Kupffer cells without contamination of other cell subsets, and thus excluded the effects of other cells such as neutrophils, monocytes and dendritic cells. Second, MoDMs of PTEN^mKO^ mice and PTEN^f/f^ mice showed no obvious differences regarding their polarization states and expression of NK cell-activating molecules (Supplementary Figures 4 and 9), suggesting that PTEN deficiency may have a less potent effect on MoDMs in the liver. Third, the number of peritoneal cavity macrophages invading into the liver was very low compared with liver resident Kupffer cells during sterile hepatic injury,^14^ and our results showed that even mice were treated by peritoneal wash (Supplementary Figure 1C), Kupffer cell depletion would lead to a significantly compromised liver regeneration rate, suggesting that Kupffer cells may be more preponderant in number and function during liver regeneration and thus may have a more important role in this process compared with other cells such as peritoneal cavity-derived macrophages and dendritic cells.

Tissue macrophages can be classified into two subsets according to their origins: MoDMs, which are derived from the bone marrow, and tissue resident macrophages, which develop from the yolk sac.^18^ Liver MoDMs and resident Kupffer cells are regulated by different transcriptomes and thus have distinct functions in a few models, such as drug-induced acute liver injury,^19^ ischemia reperfusion injury,^20^ cholestatic liver injury^21^ and liver fibrosis.^22^ Our results indicate that PTEN is of great importance to the polarization and activation of Kupffer cells, but has little effect on MoDMs (Supplementary Figures 4 and 9). Therefore, considering the heterogeneity of the liver macrophage pool, it is not surprising that the same protein has different functions.

In this study, we have also highlighted the crosstalk of Kupffer and NK cells in the liver. In fact, it has long been reported that macrophages are able to activate NK cells, either directly through cell surface markers, such as CD48&2B4,^23^ CD40&CD154 (refs 24, 25) and MICB/RAE-1& NKG2D;^26, 27^ or indirectly through cytokine mediated signals, including IL-12,^28, 29^ IL-18,^29^ IL-1*β*, IL-15 and IFN-*β*.^30, 31^ In particular, M1-polarized macrophages, but not resting or M2 macrophages, are potent activators of NK cells.^30, 31^ Indeed, we report that Kupffer cells interact with NK cells during liver regeneration. Unexpectedly, Kupffer cells stimulated the activation of NK cells in contrast to their supportive role in liver regeneration, thus hindering this process. Moreover, the critical role of NK cells in regulating Kupffer cell activity should also be highlighted. In fact, several lines of evidence have shown that NK cells are capable of modulating the biological activity of Kupffer cells. For example, in a bile duct ligation-induced murine cholestasis model, the reciprocal interaction of hepatic NK cells and Kupffer cells stimulates IL-6 production from the latter and this results in ameliorated cholestatic liver injury.^32^ In another study, NK cell-derived IFN-*γ* and Kupffer cell-derived proinflammatory cytokines, such as TNF-*α*, IL-12 and IL-18, act synergistically to mediate acute liver injury.^29^ NK cells are also capable of regulating M1/M2 polarization of Kupffer cells, promoting M1-like polarization while suppressing M2-like polarization through secreting IFN-*γ*.^19, 33, 34^ Thus, it is possible that Kupffer cell activity could in turn be affected by NK cells in our study. Considering that NK cells of PTEN^mKO^ mice produce less IFN-*γ* after PHx compared with those of PTEN^f/f^ mice (Figures 4a and c), the microenvironment in the liver of PTEN^mKO^ mice will be more favorable for the M2-like polarization of Kupffer cells. Moreover, these M2-like Kupffer cells are further compromised in their NK cell-activating capacity (Figures 5a–c). As a result, a positive feedback loop between Kupffer cells and NK cells is formed and this largely accounts for the enhanced liver regeneration rate in PTEN^mKO^ mice. To our knowledge, this is the first study to demonstrate the interaction of Kupffer cells and NK cells during liver regeneration, and highlights the role of PTEN in this process.

PHx can trigger an acute phase response that activates the immune system, including the production of inflammatory mediators to stimulate the proliferation of quiescent hepatocytes.^2^ Thus, initiation of normal liver regeneration requires inflammation. This is evidenced by the supportive role of inflammatory factors in liver regeneration, such as TNF-*α*,^35^ IL-6,^35, 36, 37, 38^ IL-17,^39^ IL-22,^40^ IL-15,^41^ and the complement system,^42^ and the inhibitory role of anti-inflammatory factors, such as TGF-*β*^43^ and IL-10.^44^ Thus, it is possible that Kupffer cells that are more M2-polarized will inhibit liver regeneration. However, improper inflammation could hinder the process of liver regeneration, as exemplified by IL-12 (ref. 45) and IFN-*γ*.^4, 5, 46^ In our results, myeloid PTEN deficiency did not affect the 'beneficial' inflammatory cytokines such as TNF-*α* and IL-6 in Kupffer cells and serum (Figure 6 and data not shown), but inhibited secretion of the 'harmful' cytokine IFN-*γ* by NK cells. This may provide an important force for promoting liver regeneration, and explains the discrepancy between the involvement of both anti-inflammatory M2-like polarization and proper inflammation in the process of liver regeneration.

It is well-established that macrophages can be divided into two polarization phenotypes, namely M1 macrophages (classically activated macrophages) and M2 macrophages (alternatively activated macrophages). LPS and IFN-*γ* induce the generation of M1 macrophages, whereas IL-4 and IL-13 are linked with M2 macrophage generation.^47, 48^ Within macrophages, transcription factors dictate and shape the phenotypes and functions of macrophages under physiological and pathophysiological conditions.^48^ Several transcription factors controlling macrophage polarization have been identified. For example, STAT1^49^ and interferon regulatory factor 5^50^ are involved in M1 macrophage polarization, whereas PI3K,^51^ STAT6,^52^ and peroxisome proliferator-activated receptor-*γ*^53, 54^ regulate M2 macrophage polarization. Moreover, it has been widely reported that PTEN is capable of negatively regulating the PI3K/Akt signaling pathway.^9, 55^ In addition, Naoko *et al.* reported that PTEN inhibition promoted Akt/*β*-catenin/FoxO1 signaling in macrophages, thus inhibiting proinflammatory cytokine secretion from macrophages.^13^ In line with these findings, our previous study showed that macrophage FoxO1 was important in promoting M1 macrophage polarization.^15^ In this study, significantly higher activation levels of Akt and lower activation levels of FoxO1 were determined in the Kupffer cells of PTEN^mKO^ mice (Supplementary Figure 6). Thus, it is possible that in PTEN^mKO^ mice, PTEN deficiency enhanced Akt/FoxO1 signaling in macrophages. As a result, proinflammatory cytokine secretion ability of macrophages was suppressed, whereas their anti-inflammatory cytokine secretion ability was promoted, leading to the M2-like polarization of these cells. Therefore, our finding that PTEN is a novel regulator of M2-like polarization of Kupffer cells is in line with and extends previous work.

Importantly, PTEN deletion not only affects the immune responses of Kupffer cells, but this also impacts growth factor secretion by these cells. Normally, a single gene modification in mouse models affects the initial regeneration rate of the liver after PHx. However, by day 10 after PHx, the process of liver regeneration will have ended and thereafter the function of a modified gene will be minimal.^2, 56^ Nevertheless, myeloid PTEN-deficient mice still demonstrate higher regeneration rates, even 10 days after PHx. Although Kupffer cell is not the only source of growth factors such as PDGF and HGF,^57, 58, 59, 60^ our results show that Kupffer cell-derived growth factors are critical for liver regeneration.

**Figure 7:**
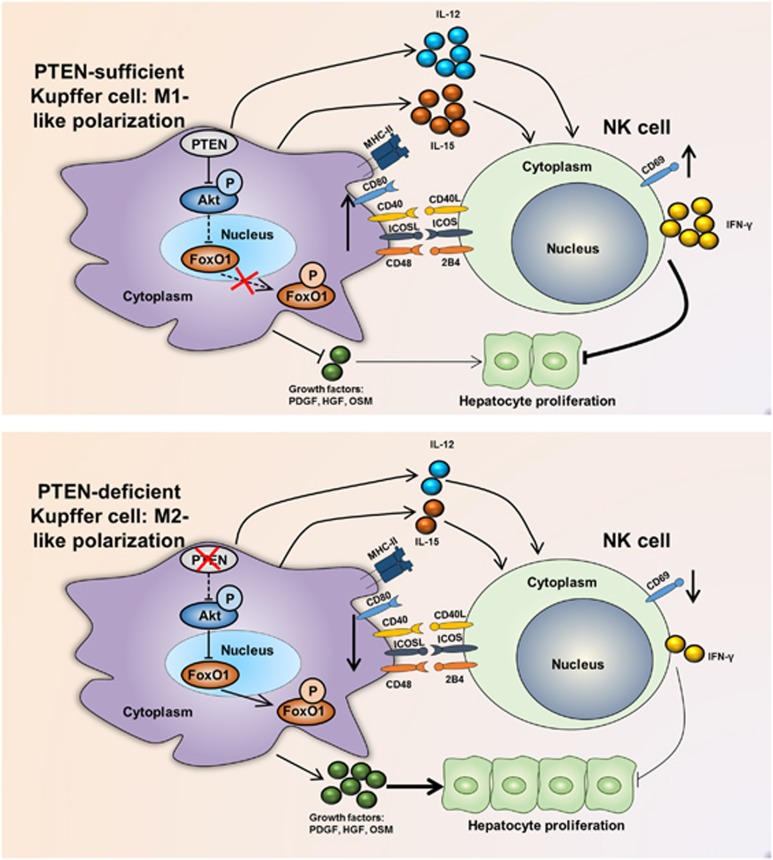
Graphic summary of this article. After PHx in PTEN-sufficient mice, PTEN expression was upregulated in Kupffer cells, which inhibited activation of Akt and thus promoted activation of downstream FoxO1 signaling, resulting in an M1-like polarization state. These M1-like Kupffer cells were more capable of activating NK cells, both through direct cell–cell contact by enhancing CD40, ICOS and CD48 signals and through facilitating secretion of IL-12 and IL-15. Consequently, NK cells in the liver were activated, and more IFN-*γ* was released, hindering hepatocyte proliferation. Moreover, Kupffer cells with higher PTEN expression levels released less growth factors such as PDGF, HGF and OSM. These effects of PTEN on Kupffer cells combined to hinder liver regeneration (upper panel). On the other hand, after PHx in PTEN-deficient mice, Akt signaling was activated and thus inhibited downstream FoxO1 signaling, resulting in an M2-like polarization state. These M2-like Kupffer cells were less capable of activating NK cells because of less expression levels of direct cell–cell contact molecules and NK cell-activating cytokines mentioned above. Consequently, NK cells in the liver were less activated, and less IFN-*γ* was released. Moreover, PTEN-deficient Kupffer cells released more growth factors mentioned above. As a result, liver regeneration rate was promoted (lower panel)

In summary, our results suggest that PTEN regulates the balance between M1 and M2 polarization of Kupffer cells during liver regeneration. PTEN deficiency resulted in an M2-like polarization of Kupffer cells after PHx, leading to the less-activated phenotype of liver NK cells, either through direct cell–cell contact or decreased IL-12 and IL-15 secretion levels. Moreover, PTEN-deficient Kupffer cells secreted more of the growth factors required for successful liver regeneration. Consequently, hepatocyte proliferation was enhanced in myeloid PTEN-deficient mice (Figure 7). Although deletion of PTEN in Kupffer cells suppressed the activation of NK cells, the ability of these Kupffer cells to secrete TNF-*α* and IL-6 remained intact. These data have therapeutic implications for liver resection.

## Materials and Methods

### Mice

C57BL/6 wild-type (WT) mice were purchased from Shanghai Laboratory Animal Center (SLAC, Shanghai, China). LysM^cre/+^ mice on a C57BL/6 background were a kind gift from Dr. Bin Gao of NIH (Bethesda, MD, USA). Mixed background loxP-flanked PTEN (PTEN^f/f^) mice were provided by Dr. Qing-Hua Shi of USTC. PTEN^f/f^ mice were backcrossed to C57BL/6 wild-type mice for at least eight generations. p40^−/−^ mice (B6.129S1-Il12btm1Jm) on a C57BL/6 J background were initially purchased from The Jackson Laboratory (Bar Harbor, ME, USA). All mice were bred in the animal center of USTC in specific pathogen-free conditions and used according to the USTC guidelines for experimental animals. Male 8- to 12-week-old mice were used in this study. Mice were anesthetized by diethyl ether inhalation. To perform 2/3 PHx, the median and left lateral lobes of the mouse liver were ligated at the stem and excised under aseptic conditions. Sham-operated mice were anesthetized and then subjected to laparotomy and wound closure only. All surgeries and sham operations were performed in the morning.

### Experimental protocol

Three experimental protocols were followed. Unless otherwise noted, mice were in groups of 4–8. First, WT mice underwent sham or PHx treatment. Twenty-four hours later, liver Kupffer cells were analyzed for PTEN levels and polarization-related markers using flow cytometry. Second, to focus on the effect of Kupffer cell PTEN on liver regeneration, PTEN^f/f^ and PTEN^mKO^ mice underwent PHx and were killed 48 h later. Then, the following experiments were performed: (a) evaluation of the liver regeneration rate using immunohistochemistry, hematoxylin and eosin (H&E) staining and measurement of liver to body weight; (b) real-time PCR and flow cytometry analysis for polarization status and markers of NK cell activation; (c) flow cytometry analysis of liver NK cells from PTEN^f/f^ and PTEN^mKO^ mice; (d) real-time PCR analysis of Kupffer cells from sham and PHx-treated PTEN^f/f^ and PTEN^mKO^ mice to detect the expression of growth factors; and (e) western blot analysis of liver tissues for Stat3 activation levels. In addition, we used an *in vitro* system to recapitulate the *in vivo* data of these experiments. In these experiments, Kupffer cells from 48 h PHx-treated PTEN^f/f^ and PTEN^mKO^ mice were sorted. Next, the sorted PTEN^f/f^ and PTEN^mKO^ Kupffer cells were co-cultured with WT liver NK cells. Forty-eight hours later, NK cells were analyzed by flow cytometry for IFN-*γ* secreting ability, and the culture supernatant was analyzed for IFN-*γ* levels. Then, the sorted PTEN^f/f^ and PTEN^mKO^ Kupffer cells were cultured for 24 h to collect conditioned medium. Finally, the conditioned medium was added to AML-12 cells, and cells were analyzed 48 h later using flow cytometry.

### Flow cytometry

Liver mononuclear cells were isolated as described.^61^ For cell surface marker staining, single-cell suspensions were first incubated first with anti-CD16/32 (Biolegend, San Diego, CA, USA) and then incubated with fluorescent antibodies. The following antibodies were used in this article and purchased from Biolegend: FITC-conjugated anti-CD40 (3-23), anti-CD3 (17A2) and anti-CD45.2 (104); PE-conjugated anti-CD80 (16-10A1) and anti-2B4 (M2b4(B6)458.1); PerCP/Cy5.5-conjugated anti-CD69 (H1.2F3), anti-CD11c (N418) and anti-ICOS (C398.4A); PE/Cy7-conjugated anti-NK1.1 (PK136); APC-conjugated anti-F4/80 (CD8A) and anti-I-A/I-E (M5-114.15.2); Alexa 647-conjugated Annexin V; APC/Cy7-conjugated anti-CD86 (GL-1) and anti-CD45.2 (104); and Pacific Blue-conjugated anti-CD3 (17A2). DAPI was purchased from BOSTER Biotechnology Company (BOSTER, Wuhan, China).

For intracellular cytokine staining, cells were stimulated with a Cell Stimulation Cocktail (plus protein transport inhibitors) (eBioscience, Santa Clara, CA, USA) for 4 h, and fixed with a fixation/permeabilization buffer kit (Biolegend). PE-conjugated anti-IFN-*γ* (XMG1.2) was purchased from Biolegend. For intracellular PTEN and CD206 staining, cells were treated with a fixation/permeabilization buffer kit (Biolegend). Anti-PTEN antibody (9188) was purchased from Cell Signaling Technology (CST, Danvers, MA, USA), Alexa 647-conjugated secondary antibody was purchased from Life Technologies (Waltham, MA, USA), and PE-conjugated anti-CD206 antibody (C068C2) was purchased from Biolegend. For Ki-67, Akt, p-Akt, FoxO1 and p-FoxO1 staining, a FoxP3/transcription factor staining buffer set (eBioscience) was used according to the manufacturer’s instructions. PE-conjugated Ki-67 (16A8) antibody was purchased from Biolegend. Akt (4691), p-Akt (4060), FoxO1 (2880) and p-FoxO1 (9461) antibodies were purchased from CST, and Alexa 647-conjugated secondary antibody was purchased from Life Technologies. For cytometric bead array (CBA), a BD CBA Mouse Inflammation Kit (BD Biosciences, Franklin Lakes, NJ, USA) was used. Data were collected using a FACSVerse flow cytometer (BD Biosciences) and analyzed with Flowjo software (Tree Star, Inc., Ashland, OR, USA).

### Kupffer cell isolation

The liver was perfused *in situ* through the portal vein with collagenase IV and brushed with a soft toothbrush to obtain single cell suspensions. A Kupffer cell-enriched cell layer was obtained using 25 and 50% Percoll (GE Healthcare, Little Chalfont, UK). For flow cytometry, the cells between 25 and 50% Percoll were counted and stained with specific antibodies. For RNA isolation, the cells between 25 and 50% Percoll were suspended in DMEM (Thermo Fisher Scientific, Waltham, MA, USA) containing 10% FBS (Millipore, Billerica, MA, USA), and transferred to a non-treated Petri dish for 2 h. After rinsing with PBS, adherent cells, which contained >95% Kupffer cells,^62, 63^ were used for further analysis.

### Kupffer cell depletion

To deplete Kupffer cells, 100 *μ*l of clodronate liposomes (Dr. Nico Van Rooijen, Amsterdam, The Netherlands) per mouse were injected intraperitoneally 48 h before partial hepatectomy surgery. Control groups were treated with the same volume of PBS-liposomes. To exclude the effects of peritoneal cavity macrophages, the peritoneal cavities of all used mice were washed with sterile PBS in a 10 ml syringe and were immediately treated with clodronate liposomes or PBS-liposomes, respectively. Twenty-four hour later, all mice had undergone 2/3 PHx.

### RNA isolation and real-time PCR

Total RNA was isolated using RNAiso Plus (Takara, Kusatsu, Japan), and a PrimeScript RT Reagent Kit (Takara) was used for reverse transcription. Real-time PCR was conducted using SYBR Premix Ex TaqTM II (Takara). Data were collected with an ABI StepOne Real-Time PCR System (Applied Biosystems, Waltham, MA, USA). The relative expression levels of analyzed genes were normalized to *gapdh* using the 2^−ΔΔct^ method. The primers used for real-time PCR are listed in Supplementary Table S1. All primer sequences were based on the PrimerBank database and Primer-BLAST from NCBI.

### Histology

For H&E staining, liver samples were fixed in 4% paraformaldehyde and embedded with paraffin. Embedded samples were cut into 4 *μ*m sections. After deparaffinization, the samples were stained with H&E for further evaluation and viewed under a light microscope. The mitotic index was calculated as the average of the ratio of mitotic hepatocytes (hepatocytes in prometaphase, metaphase and anaphase) to total hepatocytes of five randomly chosen fields. The immunohistochemical staining protocol was described previously.^64^ The primary antibodies used were: anti-PCNA (Origene, Beijing, China) and anti-Ki-67 (CST). The secondary antibodies were HRP-linked anti-rabbit IgG, or HRP-linked anti-mouse IgG (CST). To determine the Ki-67- or PCNA-positive cell numbers, Ki-67- or PCNA-positive hepatocytes were counted in five randomly chosen fields and the average count of these five fields was set as the Ki-67 or PCNA-positive cell number.

### Kupffer cell and NK cell co-culture *in vitro*

For Kupffer cell and NK cell co-culture, FACS-sorted Kupffer cells from PTEN^f/f^, PTEN^mKO^, WT or IL-12p40^−/−^ mice 48 h post PHx were co-cultured with sorted WT liver NK cells at a Kupffer: NK ratio of 1: 2 (2.5 × 10^4^ Kupffer cells with 5 × 10^4^ NK cells per well) in DMEM medium (Thermo Fisher Scientific) supplemented with 10% fetal bovine serum (Millipore) and 50 IU/ml IL-2 (Peprotech, Rocky Hill, NJ, USA) in a 96-well plate or separated by a Transwell (Corning Incorporated, Corning, NY, USA). After 48 h, NK cells and the culture supernatant were harvested. The cells were analyzed using flow cytometry for NK cell’s IFN-*γ* secreting ability analysis, and the supernatant was analyzed for IFN-*γ* concentration by CBA from a FACSVerse flow cytometer (BD Biosciences).

### Analysis of mitogenic effects of Kupffer cells to hepatocytes *in vitro*

Approximately 2 × 10^4^ PTEN^f/f^ or PTEN^mKO^ Kupffer cells (FACS sorted from 48 h PHx mice) per well were cultured in a 48-well plate in DMEM/F12 medium (Thermo Fisher Scientific) supplemented with 10% fetal bovine serum (Millipore). After 24 h, the supernatant of PTEN^f/f^ and PTEN^mKO^ Kupffer cell cultures was collected. Then, this conditioned medium was added to a 48-well plate containing 2 × 10^4^ AML-12 cells per well (AML-12 cells are a normal mouse hepatocyte cell line, and were provided by Dr. Yanyan Tao of Shuguang Hospital, Shanghai University of Traditional Chinese Medicine). After another 48 h, AML-12 cells were harvested with Trypsin-EDTA (Thermo Fisher Scientific) and counted with a FACSVerse flow cytometer (BD Biosciences).

### Western blot analysis

Frozen liver samples were homogenized with RIPA buffer (Beyotime Biotechnology, Beijing, China) supplemented with PMSF (Beyotime Biotechnology) and PhosSTOP (Roche, Basel, Switzerland). Total proteins were quantified with a Pierce BCA Protein Assay Kit (Thermo Fisher Scientific) and adjusted to a final concentration of 10 *μ*g/*μ*l. Primary antibodies for western blot analysis were as follows: anti-STAT3 (CST), anti-phospho-STAT3 (Tyr705) (CST) and anti-GAPDH (Genesci, Shanghai, China). Secondary antibodies were HRP-linked anti-rabbit IgG, or HRP-linked anti-mouse IgG (CST). Western blot data were analyzed with ImageJ software (NIH, Bethesda, MD, USA).

### Serum alanine aminotransferase (ALT) analysis

To evaluate the extent of liver injury, PTEN^f/f^ and PTEN^mKO^ mouse serum was collected at different time points of PHx, and ALT levels were measured using a commercially available kit from Rsbio (Shanghai, China).

### Statistical analysis

The data are presented as the mean±S.D. and are representative of at least two replicates. Two-tailed Student’s *t*-tests were used to identify significant differences. Differences were considered significant if the *P*-value was <0.05 (*P<0.05, **P<0.01, ***P<0.001).
