# Supplementary material for: Modulation of liver regeneration via myeloid PTEN deficiency
Source: Cell Death Dis. 2017 May 25;8(5):e2827–. doi: 10.1038/cddis.2017.47 (PMC5520744; doi:10.1038/cddis.2017.47)
Supplement: Supplementary Figure Legends and Tables [file cddis201747x1.docx]

**Supplementary Figure Legends**

**sFig. 1 Kupffer cell analysis during liver regeneration.**

(A-B) Gating strategy for Kupffer cells. Non-parenchymal cells were isolated from collagenase-digested livers. Liver resident Kupffer cells were gated as CD45^+^DAPI^-^F4/80^hi^CD11b^lo^. MoDMs were gated as CD45^+^DAPI^-^F4/80^lo^CD11b^hi^, which expressed differential levels of Ly-6c and no Ly-6G. (C) Wild-type mice were treated with PBS-liposomes (n=3) or clodronate liposomes (n=3) 48h before 2/3 PHx; 48h later, liver Kupffer cells were analyzed using flow cytometry. (D) All wild-type mice were treated with sterile PBS in the peritoneal cavity and injected immediately with PBS-liposomes (PBS, n=8) or clodronate-liposomes (Clo, n=8) 24h before PHx. The liver weight (LW) to body weight (BW) ratio was analyzed 48h post-2/3 PHx in these mice. (E) Kupffer cell numbers before (n=5) and after 2/3 PHx (n=5) were analyzed using flow cytometry.

**sFig. 2 The extent of liver injury is similar between PTEN^f/f^ mice and PTEN^mKO^ mice after 2/3 PHx.**

(A) Representative H&E. staining of liver sections at various time points post PHx from PTEN^f/f^ (n=4 for 12h, n= 4 for 24h, n=3 for 48h) and PTEN^mKO^ (n=6 for 12h, n= 4 for 24h, n=4 for 48h) mice. (B) Serum ALT analysis of PTEN^f/f^ (n=3 for sham, n=5 for 12h, n= 5 for 24h, n=3 for 48h) and PTEN^mKO^ (n=4 for sham, n=6 for 12h, n= 4 for 24h, n=3 for 48h) mice at various time points post PHx.

**sFig. 3 Representative H&E staining of liver sections showing mitotic hepatocytes.**

(A) Representative H&E staining of liver sections showing hepatocytes in prometaphase, metaphase, and anaphase stage.

**sFig. 4 MoDM polarization is not affected by PTEN deficiency before or after PHx.**

(A) Analysis of M2-related (CD206) and M1-related (CD11c) markers of liver MoDMs in sham (left panel) and 48h PHx operated PTEN^f/f^ (n=6 for sham, n=6 for 48h) and PTEN^mKO^ (n=6 for sham, n=4 for 48h) mice (right panel) by flow cytometry.

**sFig. 5 PTEN-deficient Kupffer cells show an M2-like polarization state in steady-state.**

(A) Analysis of M2-related (*Ym-1*, *Cd206*, *Arginase-1*, and *Fizz-1*) and M1-related (*Inos* and *Il-12p40*) markers in collagenase perfused Kupffer cells from sham-operated PTEN^f/f^ (n=6) and PTEN^mKO^ (n=5) mice using real-time PCR. (B) Analysis of CD206 and CD11c expression levels of Kupffer cells in sham-operated PTEN^f/f^ (n=5) and PTEN^mKO^ (n=5) mice using flow cytometry.

**sFig. 6 PTEN regulated Kupffer cell polarization through Akt/FoxO1 signaling pathway.**

(A) Total Akt (n=3 for PTEN^f/f^ mice and n=3 for PTEN^mKO^ mice) and phosphorylated Akt (p-Akt, n=3 for PTEN^f/f^ mice and n=4 for PTEN^mKO^ mice) levels in Kupffer cells of PTEN^f/f^ and PTEN^mKO^  mice analyzed by flow cytometry 24h post PHx. (B) Total FoxO1 (n=5 for PTEN^f/f^ mice and n=5 for PTEN^mKO^ mice) and phosphorylated FoxO1 (p-FoxO1, n=5 for PTEN^f/f^ mice and n=4 for PTEN^mKO^ mice) levels in Kupffer cells of PTEN^f/f^ and PTEN^mKO^  mice analyzed by flow cytometry 24h post PHx.

**sFig. 7 NK cell activation is similar between PTEN^f/f^ mice and PTEN^mKO^ mice in steady-state.**

(A) Total liver lymphocytes were isolated from sham operated PTEN^f/f^ (n=6) and PTEN^mKO^ (n=4) mice, and treated with a PMA/ionomycin cocktail for 4h. The fraction of IFN-γ-positive NK cells (CD3^-^NK1.1^+^) was analyzed using flow cytometry. (B) The MFI of CD69, ICOS, and 2B4 on NK cells from sham operated PTEN^f/f^ (n=4) and PTEN^mKO^ (n=4) mice was analyzed using flow cytometry.

**sFig. 8 NK cell proliferation and apoptosis profiles are similar between PTEN^f/f^ mice and PTEN^mKO^ mice 48h after PHx.**

(A) NK cell percentage in total lymphocytes 48h after 3/2 PHX in PTEN^f/f^ and PTEN^mKO^ mice. (B) NK cell number of the remnant liver lobules 48h after 2/3 PHX in PTEN^f/f^ and PTEN^mKO^ mice. (C) Mean fluorescence intensity (MFI) of Ki-67 of NK cells from PTEN^f/f^ and PTEN^mKO^ mice 48h after 2/3 PHX, as analyzed by flow cytometry. (D) Annexin V and DAPI profile of NK cells from PTEN^f/f^ and PTEN^mKO^ mice 48h after 2/3 PHX, as analyzed by flow cytometry. Annexin V^+^ cells were regarded as apoptotic cells, and DAPI^+^ cells were regarded as dead cells.

**sFig. 9 The expression of NK cell activating factors is not affected by PTEN deficiency in MoDMs after PHx.**

(A) Livers of PTEN^f/f^ (n=6) and PTEN^mKO^ (n=4) mice 48h post-PHx were perfused by collagenase *in situ*, and liver MoDMs were analyzed for the expression of MHCII, CD80, CD86, and CD40 using flow cytometry.

**sFig. 10 Both direct cell-cell contact and indirect cytokine secretion are necessary for Kupffer cell-mediated effects on NK cells.**

(A) WT NK cells were co-cultured with Kupffer cells from PTEN^f/f^ (n=4) and PTEN^mKO^ (n=4) mice either directly or separated by Transwell plates for 48h, IFN-γ-secreting ability of NK cells were determined using flow cytometry. (B) WT NK cells were co-cultured with Kupffer cells from WT (n=5) and p40^-/-^ (n=4) mice for 48h, IFN-γ-secreting ability of NK cells were determined using flow cytometry.

**Supplementary Table 1 Primers used for real-time PCR in this article.**

| **Gene name** | **Forward 5′-3′** | **Reverse 5′-3′** |
| --- | --- | --- |
| *Ym-1* | CAGGTCTGGCAATTCTTCTGAA | GTCTTGCTCATGTGTGTAAGTGA |
| *Cd206* | CTCTGTTCAGCTATTGGACGC | CGGAATTTCTGGGATTCAGCTTC |
| *Arginase-1* | TGTCCCTAATGACAGCTCCTT | GCATCCACCCAAATGACACAT |
| *Fizz-1* | CCAATCCAGCTAACTATCCCTCC | ACCCAGTAGCAGTCATCCCA |
| *Inos* | GTTCTCAGCCCAACAATACAAGA | GTGGACGGGTCGATGTCAC |
| *Il-12p40* | GGAAGCACGGCAGCAGAATAA | CTTGAGGGAGAAGTAGGAATG |
| *Il-15* | CATCCATCTCGTGCTACTTGTGTT | CATCTATCCAGTTGGCCTCTGTTT |
| *Il-18* | CAGGCCTGACATCTTCTGCAA | TCTGACATGGCAGCCATTGT |
| *Pdgf-α* | GGAGATCCTTCGAGGAGCACTT | GGCGATTTAGCAGCAGATATAAGAA |
| *Pdgf-β* | TGAAATGCTGAGCGACCAC | AGCTTTCCAACTCGACTCC |
| *Hgf* | CATTGGTAAAGGAGGCAGCTATAAA | GGATTTCGACAGTAGTTTTCCTGTAGG |
| *Osm* | GCAGCTGTGGCTTTCTCTGG | TCGTCCCATTCCCTGAAGAC |
| *Vegf* | AGCAGAAGTCCCATGAAGTGA | ATGTCCACCAGGGTCTCAAT |
| *Il-6* | CTGCAAGAGACTTCCATCCAG | AGTGGTATAGACAGGTCTGTTGG |
| *Tnf-α* | AAGCCTGTAGCCCACGTCGTA | AGGTACAACCCATCGGCTGG |
| *Gapdh* | AAATGGTGAAGGTCGGTGTGAAC | CAACAATCTCCACTTTGCCACTG |
