# Supplementary figures and images for: Modulation of liver regeneration via myeloid PTEN deficiency
Source: Cell Death Dis. 2017 May 25;8(5):e2827–. doi: 10.1038/cddis.2017.47 (PMC5520744; doi:10.1038/cddis.2017.47)

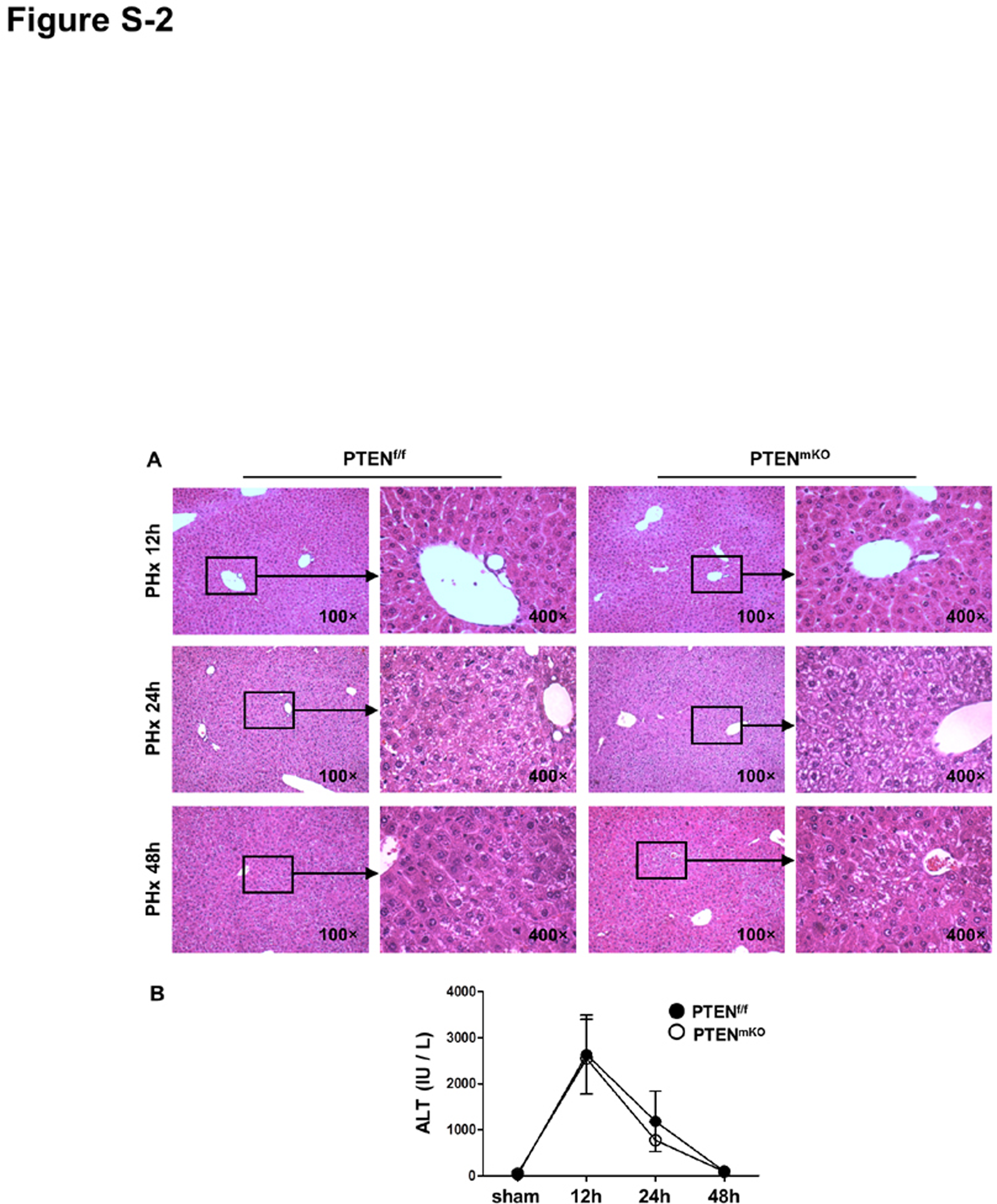

Supplement: Supplementary Figure 2 [file cddis201747x3.tif]

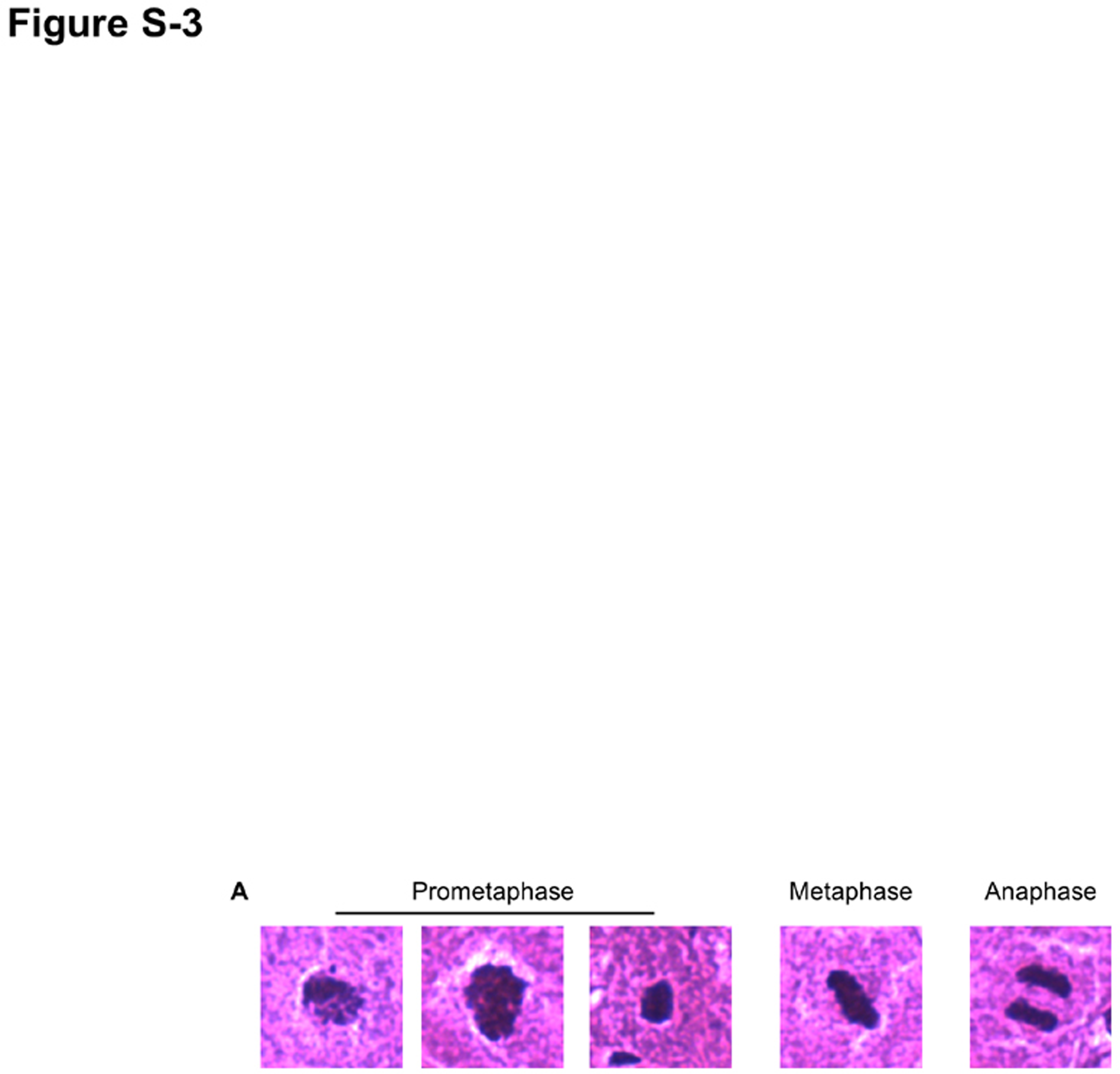

Supplement: Supplementary Figure 3 [file cddis201747x4.tif]

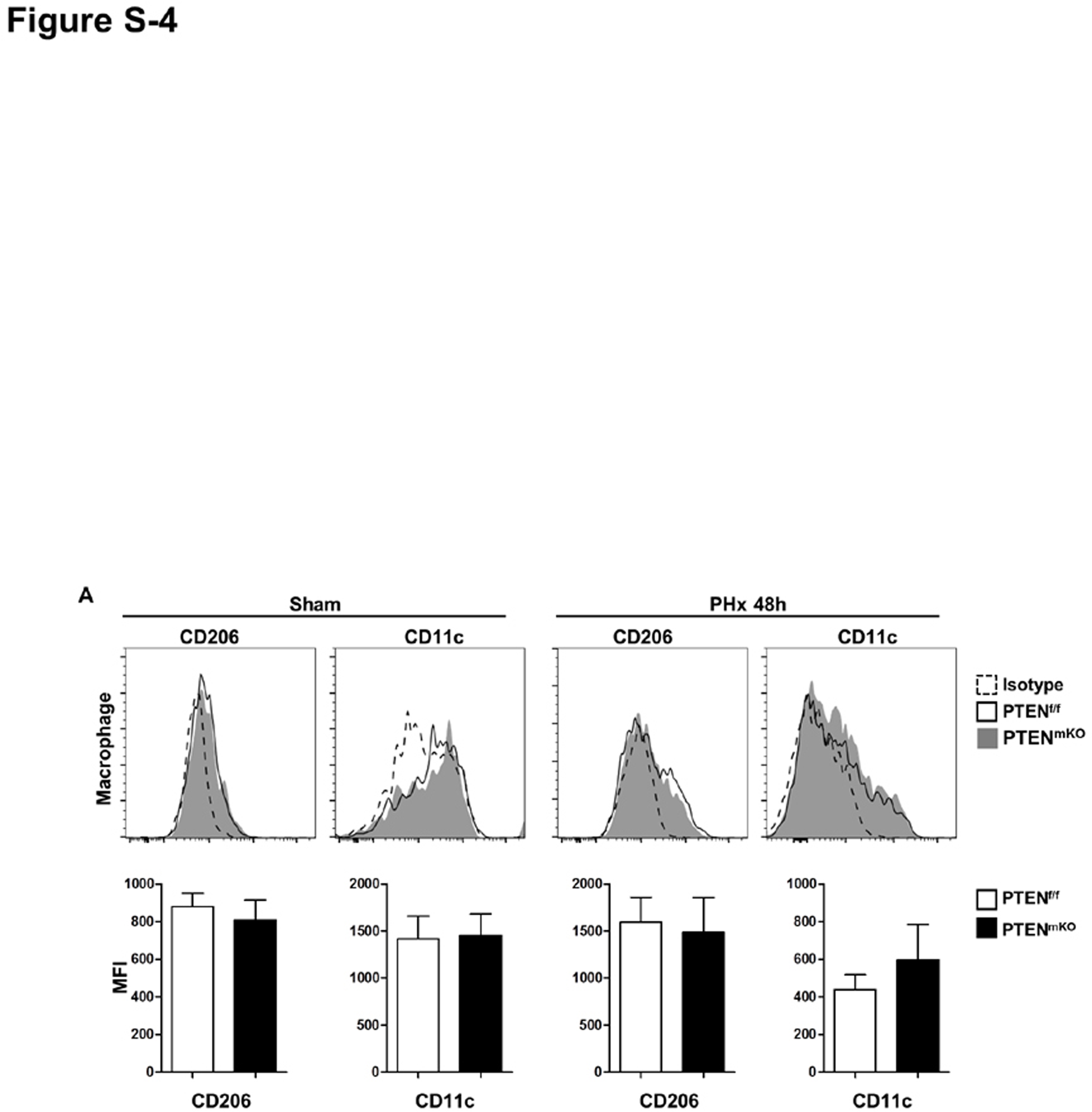

Supplement: Supplementary Figure 4 [file cddis201747x5.tif]

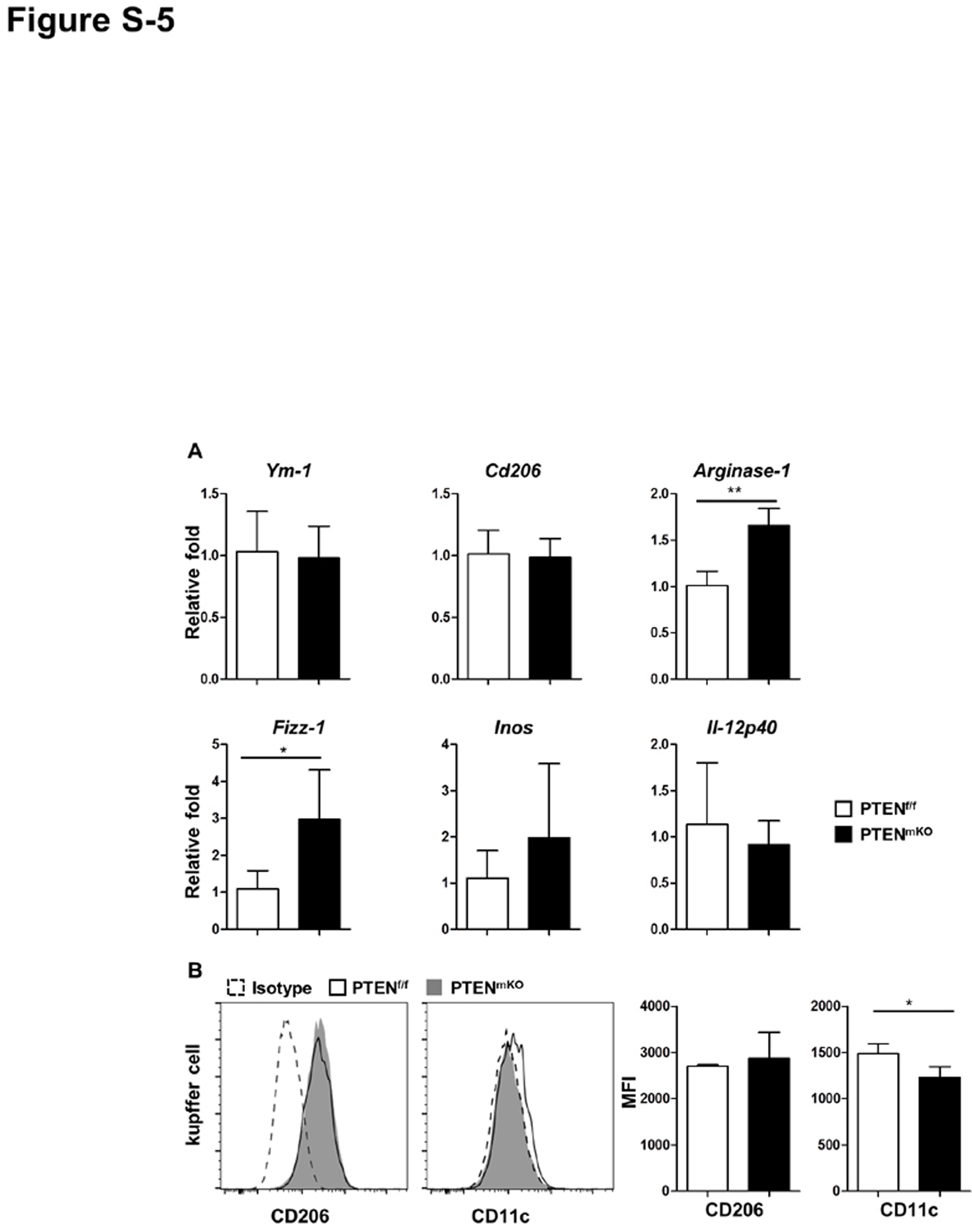

Supplement: Supplementary Figure 5 [file cddis201747x6.tif]

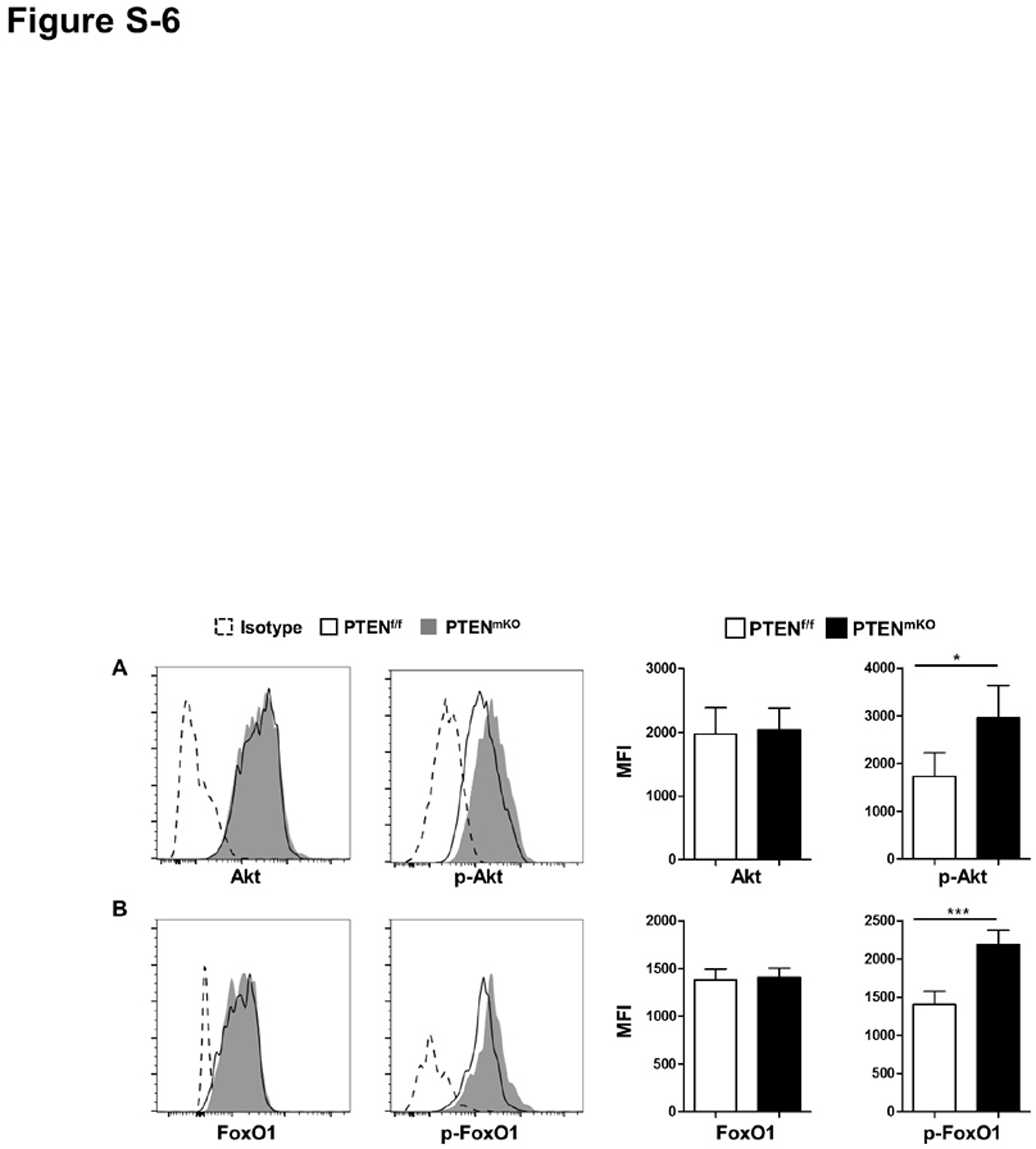

Supplement: Supplementary Figure 6 [file cddis201747x7.tif]

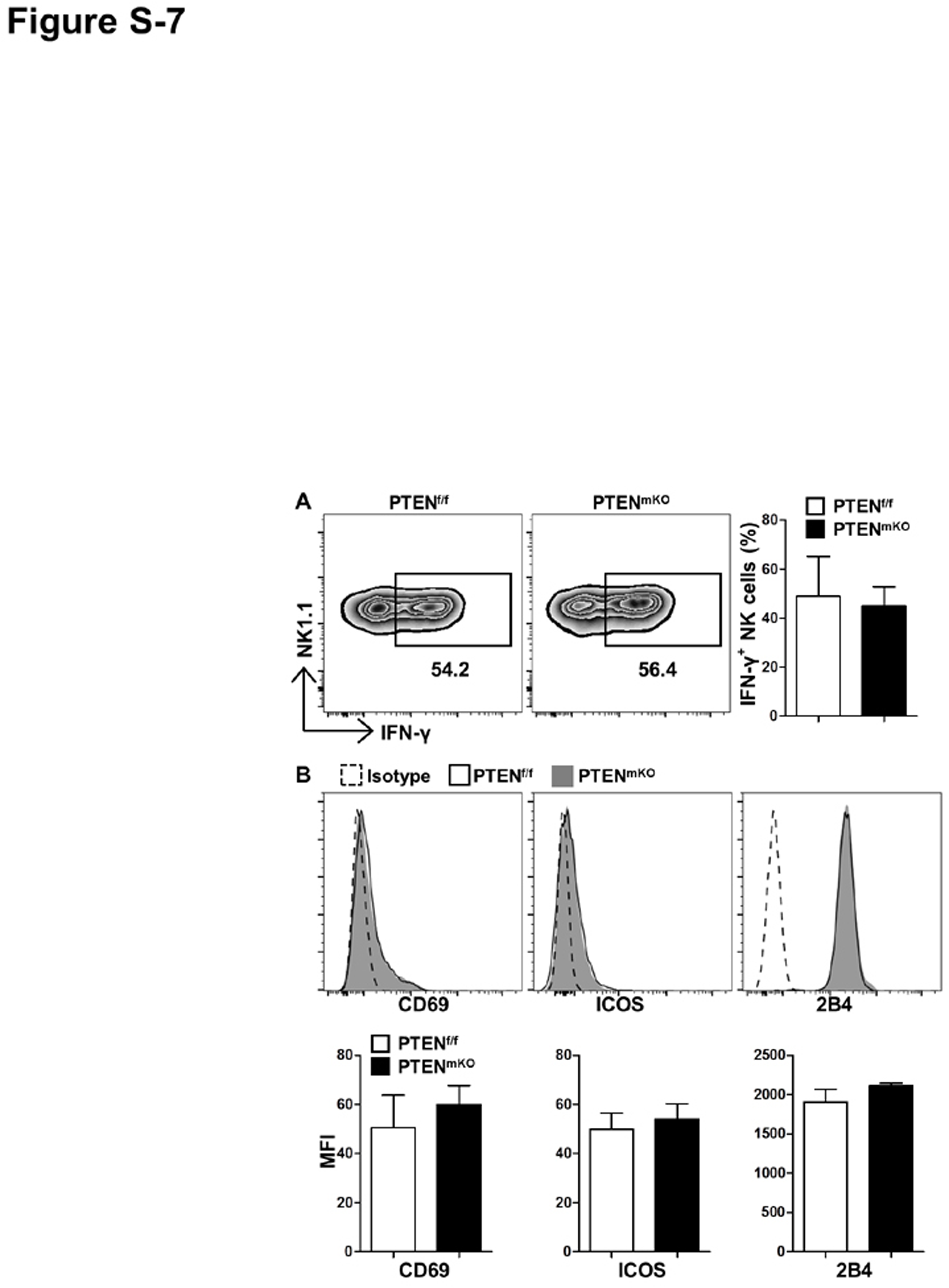

Supplement: Supplementary Figure 7 [file cddis201747x8.tif]

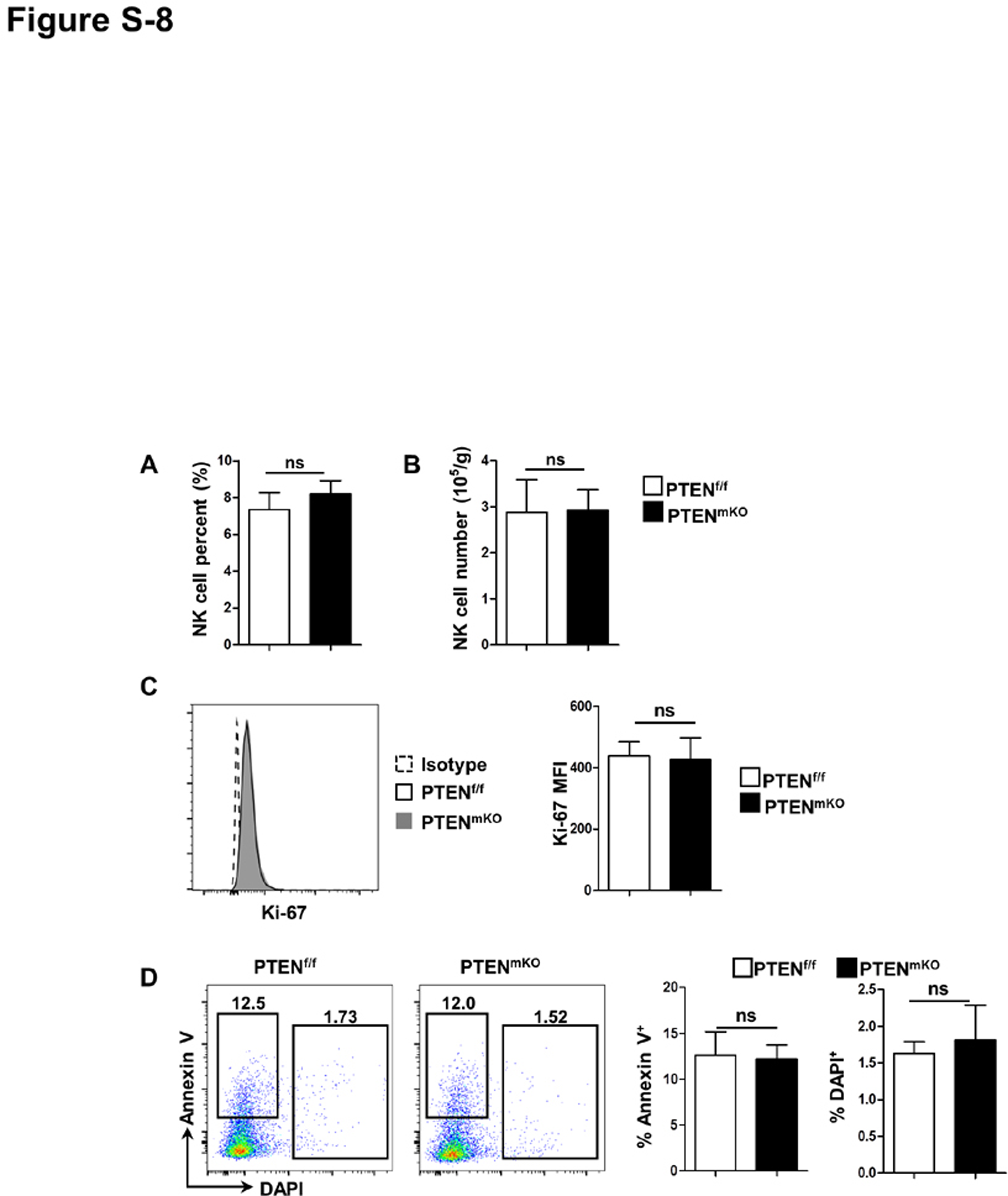

Supplement: Supplementary Figure 8 [file cddis201747x9.tif]
